# Data on antibiotic resistance among indoor microbiome at Meerut, India

**DOI:** 10.6026/97320630018293

**Published:** 2022-03-31

**Authors:** Poonam Rani, Nouratan Singh, Monika Bhaskar, Neeraj Tandan

**Affiliations:** 1 Zoology Department, Hansraj College, University of Delhi, India; 2 Department of Physiology, UPUMS, Saifai, Etawah- 206130, Uttar Pradesh, India; 3 School of Life Sciences, Department of Botany, Guru Ghasidas Vishwavidyalaya, Bilaspur - 495009, Chhattisgarh, India; 4 Scientific and Applied Research Center, Post Box No. 2241, Meerut - 250001, U.P., India

**Keywords:** House-hold microbial diversity, bacteria, fungi, antibiotic resistance, plasmid

## Abstract

Microbial dynamics of the domestic environment and their antibiotic-resistant properties have been poorly characterized. We surveyed the microbial community and their antibiotic profiling located in the rural and urban areas of Meerut city, Uttar Pradesh, India. Results show that bacterial community load across all samples had more than 100-fold higher than fungal community (all p<0.05.). Based on population load, the kitchen of both rural (Fungal: 4.16±1.81 vs Bacteria: 160.5±27.13) and urban areas (Fungal: 6.2±1.02 vs Bacteria: 205.46±30.9) were more contaminated than living rooms (rural area-Fungal: 2.13±0.74 vs Bacteria: 62.17±20.68 and urban area- Fungal: 4.75±1.68 vs Bacteria: 74.88±7.53). Six bacteria, namely *Pseudomonas* sps; *Citrobacter* sps; *Bacillus Subtilis*; Brevundimonas diminuta; Bacillus megaterium; and *Klebsiella* pneumonia, showed dominance on all other bacterial and fungal sp hence, only these six bacteria were subjected to antibiotic sensitivity test (AST). In AST, *Klebsiella* pneumoniae, *Bacillus Subtilis*, Bacillus megaterium, were resistant to more than three antibiotics. The most sensitive strain for Ciprofloxacin, Streptomycin was *Citrobacter* sp. However, *Pseudomonas* sp was found sensitive only to Amoxillin. Brevundimonas diminuta is found most sensitive to all antibiotics. Plasmid profiling of selected bacteria suggests that antibiotic resistance properties arose from plasmids, not genomic ones. These findings give new insights into the local-scale distribution of MDR bacteria in a household environment.

## Background:

With the rapid growth of population and urbanization, humans spend almost 90% of their daily lives, and this proportion can be much higher for the elderly and children. Our home is a typical representation of indoor/internal environments, thought of as a complex ecosystem that comprises many organisms, including billions of microbes [1]. Indoor microbes can adversely influence human health by various types of mechanisms such as pathogen (e.g. Streptococcus sp.) or functioning of inducements of asthma and allergies symptoms (e.g. Alternaria sp.) [2]. A range of studies have suggested that microbial diversity inside the house could partially account for the rise of chorionic inflammatory and allergies in many developed countries [3,4&^5^]. Hence, exploring the microbial diversity inside the home is of vital importance for human health and wellbeing [5]. The microbiome that resides in a home is thought to be a complex system based on various aspects, including the lifestyle of occupants and the pressure they build upon themselves, and the dispersal of the biome outside [6]. It appears that the indoor microbial diversity is related to the micro biota of human and non-human inhabitants, mostly through skin surface touch or/and direct emission of microbial particulates, and the number, gender, and behaviour of human occupants possibly will affect the microbial diversity inside the home somewhat, the attributes of the house itself such as ventilation type, design, and humidity have been reported to shape the microbiome found inside house to some extent [7]. According to the World Health Organisation (WHO, 2014) development of antibiotic resistance in different pathogenic bacteria resulting from abuse and misuse of antibiotics has been regulated as an emerging threat to modern public health [8]. The scientists investigated; certain bacterial strains that resist several drugs known as multi drug-resistant bacteria [9]. The existence of multi drug resistance in bacterial strains suggests the presence of the plasmid. Acquisition of these plasmids occurs through all three types of recombination (conjugation, transformation, and viral transduction), although conjugation appears to be the most common method for in vivo transfer [10]. Therefore, it is of interest to check the antibiotic resistance pattern of dominated micro organisms acquired either by chromosomal mutations or transferable genes mainly carried by plasmids.

## Material and methods:

### Study area and sample collection:

Indoor samples of rural and urban areas were collected from volunteers from 120 households in Meerut city, Uttar Pradesh lies approx 70 km northwest of the national capital of India (August and September 2018). Each participant was made aware of the nature of the study and supplied with sampling tools. The kitchen and living room were chosen as sampling locations as it exists in almost all houses. Air sampling in the studied area were collected for 4 minutes using the Bio Stage Impactor Quicktake pump (SKC Inc, USA) with a fixed airflow rate of 28.2 L/Minute.

### Evaluation of Microbial load of the study area:

The total fungal and bacterial count was obtained on Potato Dextrose Agar and nutrient agar plates. Fungal and bacterial colonies were expressed in terms of colony-forming units. The following formula is used for the calculation of the colony-forming unit:

Microbial load (Cfu/m³) = (Total colony x [10]³)/ (Air flow rate (28.2) x collection time in minute)

### Identification of dominant microbes:

The identification of microbes was done by classical biochemical methods described in Cowan & Steel's Manual for the Identification of Medical Bacteria [11].

### Antibiotic sensitivity test:

Six bacteria namely *Pseudomonas* sps; *Citrobacter* sps *Bacillus Subtilis*; *Brevundimonasdiminuta*; Bacillus sp; and *Klebsiella* pneumonia were showed dominance on all other bacterial and fungal sp. Hence, only these six bacteria were subjected to antibiotic susceptibility tests. The antibiotic resistance pattern of selected bacterial strains to different antibiotics was tested through the classical disk diffusion technique following Kahlmeter, 2003 [12]. Selected antibiotics and concentrations used were (µɡ/disc): Gentamycin (30), Norflaxin (10), Amoxillin (10), Vanomycin (10), Penicillin (10), Streptomycin (25), Ciproflaxin (30), Neomycin (30). The results were interpreted as per recommended guideline of the National Committee for clinical laboratory standards (NCCLS) [13].

### Extraction of the plasmid of selected microbes:

The selected bacterial isolates were screened for R-plasmid by alkaline lysis method which described in Feliciello and Chinali, 1993 [14]. A 30 µl plasmid sample was electrophoresed through 0.7% Agarose (Type 1, Sigma) with ethidium bromide (0.6µg/ml) in TE buffer at 120 V for 3 hours. The bands were visualized in a gel documentation system (Vilber Lourmat, France).

### Statistical analysis:

The two-tailed p<0.05 or listed p values were set for all statistical analysis. SPSS V16, IBM, USA) software was used to implement one-way ANOVA followed by post hock Tukey or LSD test and calculating mean and standard deviations of continuous variables.

## Result and Discussion:

### Distribution pattern of bacterial and fungal colonies isolated from the study area:

The distribution of total 120 samples for bacteria and fungi is shown in Figure 1(see PDF). A post hoc analysis showed that bacterial community across all samples had more than 150-fold higher load than fungal community (p<0.05.). On the basis, of overall diversity as well as population load, the kitchen of both rural (Fungal: 4.16 ± 1.81 vs Bacteria: 160.5 ± 27.13) and urban areas (Fungal: 6.2 ± 1.02 vs Bacteria: 205.46 ± 30.9) were more contaminated than living rooms (rural area-Fungal: 2.13±0.74 vs Bacteria: 62.17 ± 20.68 and urban area- Fungal: 4.75 ± 1.68 vs Bacteria: 74.88 ± 7.53) (all p<0.05) (Figure 1 - see PDF). In kitchen stove knobs, refrigerator handles were more contaminated as they were frequently touched by unwashed hands during cleaning of raw food. This is why home kitchens were possible places for the growth and spread of many types of microbes, including Enterococcus; streptobacillus; *Klebsiella* pneumonia; Bacillus sp. and Fungus sp. For this reason, a higher incidence of pathogens was found in the kitchen area than in the living room. In this study, the bacterial load was significantly less contaminated than in urban kitchens (Rural: 160.5±27.21 vs Urban 205±30.8; p<0.05). This variation was found to be null in the case of fungal load (p>0.05) (Figure 1 - see PDF). In fact, the kitchen in the rural area is more airy as compared to the urban area. Therefore, the load of microorganisms in the kitchen of the urban area is high. This result contrasted with those of Tyagi and Tyagi, 2013, who compared bacterial loads in rural and urban kitchens [15]. He observed that 97 % of the kitchens in rural areas are more polluted than urban kitchens. Microorganisms can survive for a long time and their survival depends on the tolerance capacity of the species and the environment. In the case of the living room, germs are easily spread by cloths and sponges during wiping [16,17]. Our results are difficult to fully interpret due to the lack of any official reference range for microbiological quality in indoor air.

### Identification of micro-organisms:

Table 1(See PDF) is showing identification of bacterial strains isolated from sampling sites. The bacterial strains are Bacillus megaterium, *Brevundimonasdiminuta*, Bacillus cereus, *Bacillus Subtilis*, *Klebsiella* pneumonia, *Citrobacter* spp, Lactobacillus, Enterococcus and *Pseudomonas* spp. In the present study, there are four major fungal genera were found in the rural and urban kitchen and living room namely *Alternaria* spp., *Mucor spp*., *Cladosporium spp.*, *Aspergillus spp*. Quality characteristics of fungal flora isolated from the air of different sections and identification were performed by lactophenol cotton blue staining (supplementary result). The bacterial community composition, Bacillus sp was the predominant species across the collected samples (Figure 2 - see PDF). Moreover, six bacteria, namely *Pseudomonas* sps, *Citrobacter* sps, *Bacillus Subtilis*, Brevundimonas diminuta, Bacillus megaterium and *Klebsiella* pneumonia, showed dominance on all other bacterial and fungal sp. Hence, out of nine, only six bacteria were selected for the further antibiotic sensitivity analysis.

### Antibiotic sensitivity pattern of selected isolates:

In this study, minimal resistance was observed for antibiotic ciprofloxacin (Ci), on the other hand, maximum resistance was found for vancomycin (Va) as shown in Table 2(see PDF). This means that most strains were sensitive to ciprofloxacin. On other hand, strains such as *Klebsiella* pneumonia, *Bacillus Subtilis* and Bacillus megaterium, were resistant to more than three antibiotics which means they are belonging to the Multidrug-resistant (MDR) category. The most sensitive strain for Ciprofloxacin, Streptomycin was *Citrobacter* sp. However, *Pseudomonas* sp was found sensitive only to Amoxillin. *Brevundimonasdiminuta* is found most sensitive to all antibiotics. MDR bacteria are a serious health problem in our country in the last few decades due to low public awareness. Environmental pollution of air, water, and soil is increasing day by day. This pollution creates selective pressure on microorganisms especially bacteria to manipulate their genes for their survival. Because of this selective pressure, bacteria can be growing, be selected, and evolved. These resistant bacteria also resist a variety of antibiotics because they have the ability to acquire and transfer resistance genes that contribute to their continued survival in an altered environment. Drug resistance is primarily acquired and transmitted horizontally through conjugation, transformation, or transduction of plasmids [18-19]. The presence of multi drug resistance among bacteria poses the most serious challenge for clinicians.

### Plasmid profiling of selected bacterial strains:

In the present study, plasmid isolation was performed to determine whether this resistance trait is genome-derived or plasmid-derived. For the estimation of the molecular weight of the plasmid, E. coli MTCC131 was used as the source of the standard plasmid marker. Macrina et al. (1978) reported that Escherichia coli (MTCC 131) harbored 8 diverse plasmids with known molecular weights i.e., 35.8 MDA, 4.8 MDA, 3.7 MDA, 3.4 MDA, 2.6 MDA, 2 MDA, 1.8 MDA, and 1.4 MDA [20]. The results from four bacteria namely Bacillus megaterium, *Klebsiella* pneumonia, *Citrobacter* sp show that the multidrug-resistant bacteria have a single plasmid of molecular weight 54.4 kb. This 54.4 kb is the same to 35.8 MDa (1 MDa=1.52 kb) of known molecular weight of plasmid of E. Coli V517 (Figure 3 - see PDF). *Pseudomonas* sp and *Bacillus Subtilis* also contain a single plasmid but have molecular weights slightly higher than 55 kb (Table 3 - see PDF). We found that most of the bacterial strains were found to be multidrug-resistant. This study was in line with Chaturvedi et al. (2008) [21]. Of all the bacterial strains, Brevundimonas diminuta was found to be the most sensitive strain. This sensitivity may be due to the absence of plasmids in Brevundimonas diminuta (Figure 3, Table 3 - see PDF). The resistance properties of Brevundimonas diminuta with little or no-showed antibiotic resistance properties were generated from plasmids. This suggests that there is a clear relationship between bacterial resistance and plasmids. These results were consistent with Shahid et al. (2003) and Oppegaard et al. (2001) [22-23]. Authors isolated a single plasmid of molecular weight 48.5 kb and 65 kb in multidrug-resistant isolates of *Pseudomonas* sp and lactose fermenting Coliform, respectively [23].

## Conclusion:

The prevalence of multidrug-resistant bacteria is quite higher than fungi in the household environment. Comparison between urban and rural samples shows that indoor microbial community load is shaped by environmental variables such as proper ventilation, sunlight, etc. Moreover, the household microbes carried a considerably high diversity of Bacillus sp and members of the Enterobacteriaceae family. Plasmid profiling of selected bacteria suggests that antibiotic resistance properties arose from plasmids, not genomic ones. Taken together, these findings provide new insights into the local-scale distribution of MDR bacteria in a household environment.

## Author contribution:

Dr. Neeraj Tandan initiated the study; Monika Bhaskar participated in the experimental design, providing sampling tools for volunteers, performing all the experiments; Neeraj Tandan wrote the first draft of this manuscript, and all authors contributed to and approved the final manuscript.

## References

[1] Amend AS (2010). Proc Natl Acad Sci U S A..

[2] Barberan A (2015). Proc Biol Sci..

[3] Rook GAW (2013). Evol Med Public Health..

[4] Mubanga M (2017). Sci Rep..

[5] Bobel TS (2018). Proc Natl Acad Sci U S A..

[6] Gilbert JA, Stephens B (2018). Nat Rev Microbiol..

[7] Gupta S (2017). Nature..

[8] Shallcross LJ (2014). The Journal of antimicrobial chemotherapy.

[9] Shiferaw T (2013). Annals of clinical microbiology and antimicrobials.

[10] Clowes RC (1972). Bacteriol Rev..

[11] Cowan ST, Steel KJ (1965). Journal of Pharmacy and Pharmacology.

[12] Kahlmeter G (2003). Journal of Antimicrobial Chemotherapy.

[13] https://clsi.org/standards/products/microbiology/documents/m100/.

[14] Feliciello I, Chinali G (1993). Analytical Biochemistry..

[15] Tyagi PK, Tyagi S (2013). African Journal of Microbiology Research.

[16] Luksamijarulkul P (2014). Oman Med J..

[17] Shen J (2014). Biomed Environ Sci..

[18] Genet C (2011). Ethiop J Health Sci..

[19] https://www.environmentaljournal.org/volume-2-issue-4.shtml.

[20] Macrina FL (1978). Plasmid..

[21] Chaturvedi S (2008). J Environ Biol..

[22] Shahid M (2003). FEMS Microbiol Lett..

[23] Oppegaard H (2001). Appl Environ Microbiol..

